# Modification of Ceramic Membranes with Carbon Compounds for Pharmaceutical Substances Removal from Water in a Filtration—Adsorption System

**DOI:** 10.3390/membranes11070481

**Published:** 2021-06-28

**Authors:** Daniel Polak, Izabela Zielińska, Maciej Szwast, Igor Kogut, Artur Małolepszy

**Affiliations:** 1 Faculty of Chemical and Process Engineering, Warsaw University of Technology, 00-645 Warsaw, Poland; daniel.polak.dokt@pw.edu.pl (D.P.); izabela.zielinska.dokt@pw.edu.pl (I.Z.); artur.malolepszy@pw.edu.pl (A.M.); 2 Doctoral School No. 1, Warsaw University of Technology, 00-661 Warsaw, Poland; 3 Hohenstein Institut für Textilinovation gGmbH, 74357 Bönnigheim, Germany; i.kogut@hohenstein.de

**Keywords:** membrane, adsorption, filtration, pharmaceuticals, PPCPs

## Abstract

The aim of this work is to develop a new type of carbon-ceramic membranes for the removal of pharmaceutical substances from water. The membranes were prepared by the chemical modification method using an organosilicon precursor—octadecyltrichlorosilane (ODTS). Graphene oxide, multi-walled carbon nanotubes with carboxylic groups, and single-walled carbon nanotubes were used in the modification process. The filtration properties and adsorption properties of the developed membranes were tested. In order to characterize the membrane, the water permeability, the change of the permeate flux in time, and the adsorbed mass of the substance were determined. Additionally, the surface properties of the membranes were characterized by contact angle measurements and porosimetry. The antibiotic tetracycline was used in the adsorption tests. Based on the results, the improved adsorption properties of the modified membrane in relation to the unmodified membrane were noticed. Novel ceramic membranes modified with MWCNT are characterized by 45.4% removal of tetracycline and permeate flux of 520 L·h·m^−2^·bar^−1^. We demonstrated the ability of modified membranes to adsorb pharmaceuticals from water streams that are in contact with the membrane. Novel membranes retain their filtration properties. Therefore, such membranes can be used in an integrated filtration–adsorption process.

## 1. Introduction

For about 20 years, there has been a growing interest in Pharmaceutical and Personal Care Products (PPCPs) that pollute the aquatic environment [1,2]. These pollutants come, to a large extent, from pharmaceutical plants and hospitals but also from agriculture (animal husbandry and plant cultivation) and households due to the widespread use of antibiotics and non-prescription drugs [3]. This accessibility and use of pharmaceuticals make these compounds appear in increasing concentrations in water and soil. The concentrations of these substances are relatively low (µg/L) [4], which makes it difficult to measure them. The problem, however, is the accumulation of the pharmaceuticals [5,6], which leads to harmful changes in the ecosystem and in the health of individuals, including humans [7,8].

Therefore, there is an urgent need to develop technologies for removing PPCPs from the environment, in particular from water. Very low concentrations of these substances and their diversity [2,9] make the development of an effective, and at the same time, universal purification method a significant challenge. Conventional methods of water purification and treatment are not efficient enough, so methods dedicated to this process are searched for in the literature. An additional difficulty in proposing an appropriate strategy is the high variability in the composition of sewage that contains PPCPs [10]. A number of methods were described for the removal of compounds of pharmaceutical origin from water. These included biological processes [11,12,13], adsorption methods [14,15,16], catalytic and photocatalytic methods [17,18,19,20], membrane methods [21,22,23], and mixed methods [24,25]. None of these methods proved to be sufficiently effective against the test substances. The right direction in finding the correct way seems to be to combine various processes to benefit from each of them.

In the literature, there are papers presenting attempts to remove PPCPs using membrane techniques. To this end, numerous authors modified membranes to make the membrane processes more effective for these substances. Cellulose acetate nanofiltration membranes modified by charged molecules were investigated [26,27] and similar modifications were made on ultrafiltration membranes made of poly(ether sulfone) [28,29]. Such modifications improved the separation properties of membranes in relation to many PPCPs type compounds. Information on nanofiltration and reverse osmosis membranes modified with graphene compounds and then used to remove pharmaceuticals can be found in the review paper [30]. Polyamide membranes were also modified with graphene oxide and the retention of PPCPs compared to unmodified membranes increased [31]. Two issues should be noted here: (i) the modification of membranes with various compounds led to the improvement of the separation properties of the membranes obtained in this way, which is also the aim of this paper; (ii) the literature concerns in particular polymer membranes, not ceramic, and applied to more precise techniques than microfiltration used in this work. However, there are also studies on the successful modification of ceramic membranes used for PPCPs removal from water [32,33]. It should be emphasized that the research in this paper focuses in particular on imparting adsorption properties to membranes, and not on improving filtration abilities, which was the purpose of the research presented in most of the studies conducted so far in the literature. In this paper, an integrated membrane adsorption process is proposed in which some substances contained in sewage would undergo retention on the membrane, while other substances would be adsorbed on the membrane surface. To this end, membranes modified by carbon compounds were proposed. This solution proved effective in our earlier research, including other sewage types [34,35]. Carbon compounds such as graphene, graphene oxide, reduced graphene oxide and carbon nanotubes have excellent adsorption properties [36,37], also for PPCP compounds [38,39,40]. The combined advantages of membrane techniques with the adsorption properties of carbon compounds helped effectively remove selected pharmaceutical compounds from water [29,41,42,43,44].

The aim of the study was to modify the surface of ceramic membranes using graphene oxide (GO), single-walled carbon nanotubes (SWCNTs) with -COOH groups and multi-walled carbon nanotubes (MWCNTs) with -COOH groups. The tests showed that ceramic membranes could be given adsorption properties by surface modification and then they could be used in an integrated membrane and adsorption process.

## 2. Materials and Methods

### 2.1. Materials

Commercially available nineteen-channel ceramic membranes Star-Sep (Mantec Filtration, Longton, UK) were used for the tests. These membranes are made of α-Al_2_O_3_; the average diameter of the pores is 0.35 µm. The following materials were used in the modification process: Octadecyltrichlorosilane (ODTS, Sigma-Aldrich, Saint Louis, MO, USA), Graphene Oxide (GO) (own production), single-walled carbon nanotubes (SWCNTs) with -COOH groups (Sigma-Aldrich, Saint Louis, MO, USA), and multi-walled carbon nanotubes (MWCNTs) with -COOH groups (Sigma-Aldrich, Saint Louis, MO, USA). Isopropanol (Sigma-Aldrich, Saint Louis, MO, USA) was used to prepare ODTS solutions, while ethanol (Sigma-Aldrich, Saint Louis, MO, USA) was used to prepare carbon compound suspensions. The antibiotics tetracycline (POL-AURA. Warsaw, Poland) was used to test the adsorptivity of the membranes in relation to pharmaceutical substances.

### 2.2. Membrane Surface Modification

**Figure 1:**
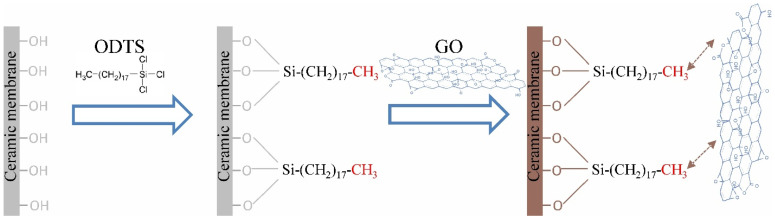
Ceramic membrane modification mechanism.

Membranes were chemically modified with anchoring compounds. ODTS played this role in the presented tests. This compound belongs to the group of silanes. These substances have self-organizing properties which help to form a monolayer on the surface of the materials. For ODTS, the bonding process consists of forming bonds between the silicon atom and the hydroxyl groups located on the membrane surface (Figure 1). Free methyl groups in the ODTS structure can be bonded with the surface of carbon compounds due to physical or chemical effects by forming bonds between them and the organic groups located on the surface of the materials. With silane compounds, a durable and stable layer is produced on the material’s surface [26].

The modification of membranes with ODTS solution in isopropanol (2 L) was carried out in the flow-through system at room temperature. The solution was pumped through the membrane module with a peristaltic pump (1 L/min); and the solution was circulated in the system for 15 min. Then the membrane was dried for 24 h, washed with ultrapure water, and dried again for the following 24 h. The membrane was placed in the carbon compounds suspension for a certain time. After the modification, the membrane was cleaned with ultrapure water again and dried in an oven at 80 °C. Furthermore, 50 cm long membranes (ca. 0.14 m^2^) were used for the modification process. The membrane samples were prepared for a porosimetric analysis, the contact angle measurement, and for process tests in the stationary and flow-through systems.

### 2.3. Membrane Adsorption Properties

The adsorption properties of the membranes were tested in the stationary and flow-through systems. In both systems, the change in the concentration of tetracycline was measured during the process. UV–Vis-spectroscopy (Gensys 10S UV-Vis, Thermo Fisher Scientific, Waltham, MA, USA) was used to determine the concentration of the substance. The samples were scanned in the range of 250–400 nm, while the peak for the tetracycline could be found at 358–360 nm. Tests in the stationary system were carried out in a beaker in which a 2.5 cm long sample membrane was placed and then 40 mL of tetracycline solution was poured. The initial concentration of the tetracycline solution was 0.033 g/dm^3^. The mass of the adsorbed substance *q_stat_*, Equation (1) was a parameter to describe the adsorption properties of the membrane after 90 min of the process duration.

(1) $$q_{stat}=\frac{\left(c_{0}-c_{90}\right)V_{s}}{S},$$

where: *q_stat_*—mass of the adsorbed substance on the membrane surface [g/m^2^], *c*_0_—initial tetracycline concentration [g/dm^3^], *c*_90_—tetracycline concentration after 90 min of the process duration [g/dm^3^], *V_s_*—volume of the tetracycline solution (0.04 dm^3^) [dm^3^], *S*—membrane surface (0.007 m^2^) [m^2^].

For tests in the stationary system, membranes modified by solutions of various ODTS concentrations and different modification times with carbon compounds were prepared. The modification process parameters of the test membranes are shown in Table 1.

On the other hand, the tests of adsorption properties in the flow-through system were carried out using a laboratory-based microfiltration installation. The installation is described later in the paper.

### 2.4. Filtration–Adsorption Setup

**Figure 2:**
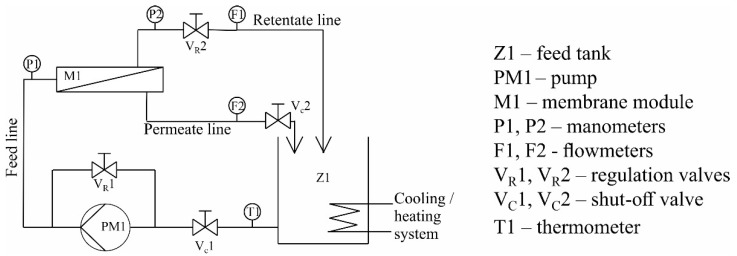
Installation scheme for microfiltration.

The tests for removing tetracycline with an integrated filtration–adsorption process were performed using a conventional microfiltration pilot plant. The installation scheme is shown in Figure 2. The tests were conducted in a closed-circuit system of the retentate and permeate. In this system, the pump PM1 pumped the tetracycline solution from the tank Z1 through the membrane module M1. Both the stream of the retentate and the stream of the permeate returned to the tank Z1. In this system, the permeate flowed from the inside to the outside of the membrane (bore side feed). In order to maintain a constant temperature of the feed the tank Z1 was thermostated. Moreover, 40 cm long membranes were used in the tests (10 cm of 50 cm modified membrane were used for other tests). The volume of tetracycline solution used with the concentration of 0.033 g/dm^3^ was 7.5 dm^3^ in the test solution. The filtration process was conducted at the transmembrane pressure of 1 bar and the feed volumetric flow of 400 dm^3^/h.

Using the system to carry out the microfiltration process, the water permeability, Equation (2), was determined and the mass removed from the tetracycline solution, Equation (3) was measured (water samples were taken from the tank Z1). The water permeability was determined for ultrapure water. In addition, the change in the volumetric flow of permeate and the change in the concentration of tetracycline in the feed and permeate stream were measured during the process.

(2) $$WP=\frac{Q_{p}}{p_{N}S},$$

where: *WP*—water permeability [dm^3^/(min·bar·m^2^)], *Q_p_*—permeate volumetric stream [dm^3^/min], *p_N_*—feed pressure [bar], *S*—membrane surface [m^2^].

(3) $$q_{flow}=\frac{\left(c_{0}-c_{90}\right)V_{s}}{S},$$

where: *q_flow_*—mass of the adsorbed substance on the membrane surface [g/m^2^], *c*_0_—initial tetracycline concentration [g/dm^3^], *c*_90_—tetracycline concentration after 90 min of the process duration [g/dm^3^], *V*_s_—volume of tetracycline solution (7.5 dm^3^) [dm^3^], *S*—membrane surface (0.11 m^2^) [m^2^].

Based on the experience of the previous tests carried out in the stationary system, eight different membranes were prepared for tests in the flow-through system. The modification process parameters for individual membranes are shown in Table 2.

### 2.5. Examination of Membrane Surface and Structural Properties

The wettability of modified membranes were examined by the contact angle. If the contact angle is lower than 90°, the membrane surface is hydrophilic. In water filtration, it is better to use more hydrophilic membranes. That is why these tests were conducted. The sessile drop method was used to determine the contact angle of the membrane surfaces. The procedure was performed with the goniometer OCA 25 DataPhysics. The tests were carried out using ultrapure water with a drop volume of 0.5 µL. Each sample was tested at five random locations, and then an average value was determined.

The flow porosimeter PMI Capillary iPore-1200A was used to determine the pore diameter distribution of the membranes being produced. Each sample was tested three times. The sample was tested in the range of gas pressure from 0 to 7 bar. A substance with a surface tension of 15.9 mN/m was used as wetting liquid.

## 3. Results and Discussion

**Figure 3:**
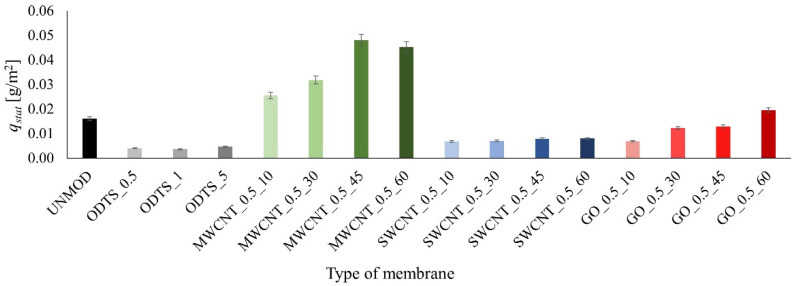
Mass of the adsorbed tetracycline in the stationary system.

The first part of the tests determined the effect of the presence of ODTS and carbon compounds on the adsorption properties of the membranes. To this end, membranes modified by ODTS solutions in isopropanol with various concentrations, ranging from 0.5%, 1%, and 5% were prepared. The modification took 15 min. Such time was sufficient to ensure even coverage of the membrane surfaces. Then, membranes modified by both ODTS and carbon compounds were prepared. The concentration of the suspensions used was 0.04% in ethanol. The use of low concentrations reduces the risk of membrane clogging as a result of the creation and deposition of agglomerates in the pores. Therefore, higher concentrations were not used in the tests. The effect of the membrane modification time on their adsorption properties was checked. The obtained values of the adsorbed tetracycline mass *q_stat_* after 90 min of the process for the test membranes are shown in Figure 3.

**Figure 4:**
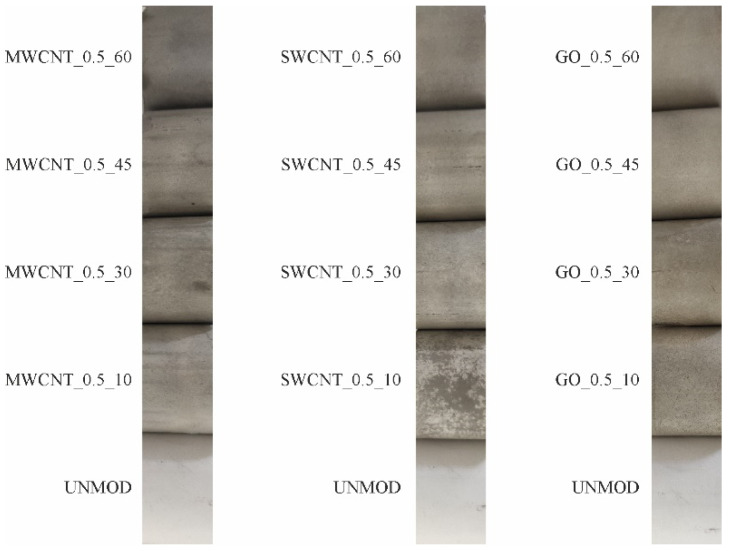
Images of the test membrane samples. Photo taken by 12MPx camera without magnification.

On the basis of the results, it can be concluded that the presence of MWCNTs and GO improved the adsorption properties of ceramic membranes. In addition, it should be stressed that when GO was used, the properties improved only after a long time of modification, i.e., 60 min of the process duration. There is also a trend towards an increase in the amount of adsorbed tetracycline as the membrane modification in a carbon suspension proceeds. This results from the fact that as the modification time proceeds, the number of carbon particles inoculated on the membrane surface increases. This is confirmed by the analysis of membrane sample images, as shown in Figure 4. As the modification time proceeds, the intensity and uniformity of the membrane color increase as well.

**Figure 5:**
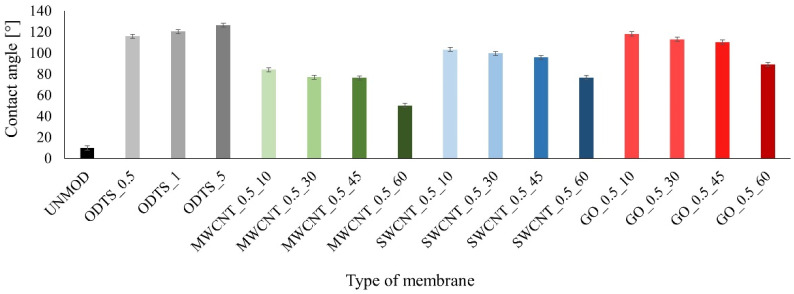
Results of the contact angle measurement for the test membranes.

The parameter that confirms that more carbon particles are deposited on the membrane surface is the contact angle which is significantly higher than for the unmodified membrane. In addition, the contact angle decreases as the modification phase in the carbon compound suspension proceeds. The values of the measured contact angles are presented in Figure 5. Contact angle values for modified membranes are higher than for the unmodified membrane for two reasons. Firstly, there are free organic chains originating from ODTS molecules on the surface of the modified membranes and they are hydrophobic in nature. Secondly, the carbon compounds used have lower hydrophilic properties than unmodified ceramics.

The differences between the values of the contact angles between membranes modified by various carbon compounds may result from the properties of the compounds themselves and the amount inoculated on the membrane surface.

In addition, based on the results of the adsorbed substance mass and the contact angle, it can be noted that for membranes that are only ODTS-modified, i.e., having a high contact angle, there was an evident deterioration in the adsorption properties. On the other hand, the MWCNT-modified membranes, which are those with the best wettability among the modified membranes, have the best adsorption properties. On this basis, it can be argued that the amount of the adsorbed substance depends both on the specific chemical effects between the organic groups on the surface of the carbon compounds and the organic groups in the structure of the pharmaceutical substance and on the surface effects. For the test tetracycline, the degree of its adsorption will be low for low wettability materials. On the other hand, the assumption that there is a possibility of specific effects between the adsorbing material and the pharmaceutical substance is confirmed by the results for the GO-modified membranes. These membranes have better adsorption properties despite a higher contact angle than the SWCNT membranes. The paper presents the results of the SWCNT-modified membranes, although they have weaker adsorption properties than unmodified membranes. However, it should be stressed that, hypothetically, the group of pharmaceutical compounds includes substances with diverse chemical structure. For this reason, SWCNTs may prove to be a better adsorbent for compounds with different properties than tetracycline. In addition, the results of the adsorbed substance mass and the contact angle indicate that the presence of SWCNTs altered the properties of the ceramic membranes. On this basis, it can be concluded that this carbon compound can also be used to modify ceramic membranes with a method based on anchoring compounds.

For the tests in the flow-through system, an unmodified membrane was selected, a membrane modified with 0.5% ODTS solution and membranes modified with 0.5% ODTS solution, and then modified with carbon compounds for 60 min. The 60-min modification time provided the best adsorption properties for the membranes being produced. In addition, there were membranes modified with 1% ODTS solution prepared, which were modified for 60 min with carbon compounds to check the effect of the ODTS solution concentration on the membrane properties. Further increasing of the ODTS solution concentration caused a significant decrease in the *WP* value. The *WP* value for the membrane modified with 5% ODTS solution was 2.8 dm^3^/(min∙bar∙m^2^), whereas for the unmodified membrane it is 24.0 dm^3^/(min∙bar∙m^2^). This is probably due to the pores being covered with ODTS molecules during the process of modification in the flow-through system. In addition, this compound has strong hydrophobic properties, which further reduced the permeability of the membrane. From the point of view of the filtration process, a clear decrease in the permeability of the membranes is unfavorable; therefore, this membrane was rejected from further testing.

**Figure 6:**
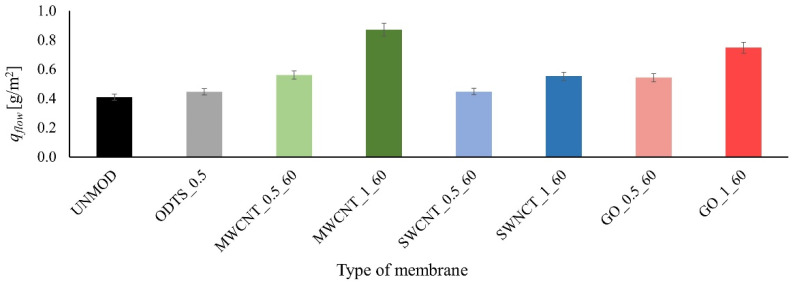
Mass of the tetracycline removed from water in the flow-through system.

The results of the mass of the removed tetracycline from water *q_flow_* obtained in the flow-through system are shown in Figure 6.

Based on the results, it can be concluded that modified membranes are more adsorptive than unmodified membranes. The most significant improvement was achieved for MWCNT-coated membranes. In addition, membranes modified with 1% ODTS solution provided a higher degree of drug removal from water than membranes modified with 0.5% solution. This is due to the higher amount of ODTS that covered the membrane surface, which led to the increase in the number of inoculated carbon compound particles. One of the reasons for the differences in the amount of tetracycline removed with the use of a given membrane is that they vary in terms of adsorption properties, as already demonstrated in the tests in the stationary system.

Table 3 shows the results of the measurements of tetracycline concentrations in permeate and retentate during the filtration process. It can be noted that at a given point in time, the concentrations in permeate *c_P_* and retentate *c_N_* are equal. This is understandable if we compare the size of the tetracycline molecule and the membrane pore size. However, the decreasing tetracycline concentration in these two streams proved that the substance was adsorbed on the membrane’s surface or in its pores.

**Figure 7:**
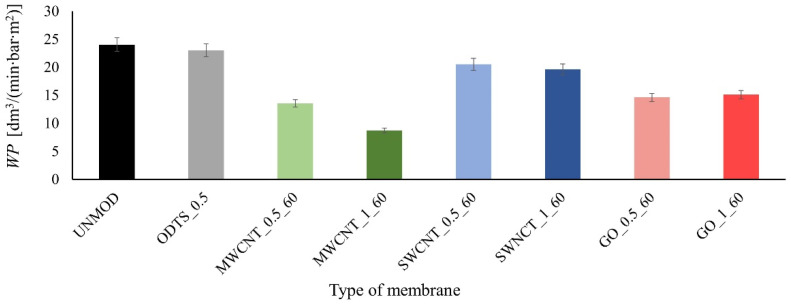
Value of the water permeability *WP* in the test membranes.

**Figure 8:**
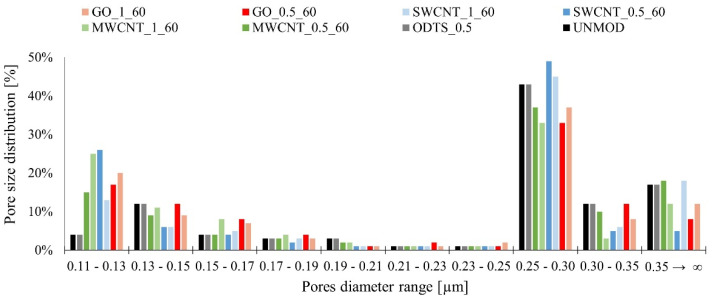
Pore diameter distribution for the tested membranes.

Using modified membranes in an integrated filtration–adsorption process and the adsorption properties discussed earlier, the filtration properties of the membranes are also important. These properties, described by the water permeability *WP* and the pore diameter distribution, can be improved or deteriorated during the modification process. The results of the water permeability measurements are shown in Figure 7, while the pore diameter distribution is shown in Figure 8.

On the basis of the obtained *WP* values, a deterioration in the permeability of the modified membranes by carbon compounds may be observed for the unmodified membrane and the membrane modified only with ODTS. Additionally, it should be noted that the decrease in *WP* is greater for membranes modified with 1% solution and the lowest values were obtained for MWCNT coated membranes. On the basis of the results of the mass of the removed tetracycline and the *WP* value, it can be concluded that as the coating intensity of the membrane increases, which depends on the concentration of the modifying solution used and the type of the carbon compound used, the permeability of the membrane decreases and the mass of the removed tetracycline from water increases. This is probably due to the deposition of ODTS molecules and carbon materials not only on the surface of the membrane but also in its pores. This is further confirmed by the results of the porosimetric analysis. However, it should be stressed that the *WP* values for modified membranes are still acceptable in filtration processes.

On the basis of the porosimetric analysis, it can be concluded that for membranes coated with carbon compounds, the percentage share of pores with a smaller diameter increases, particularly with a diameter range of 0.11 µm to 0.13 µm as compared to the unmodified membrane and a membrane modified only with ODTS. This is due to a reduction in the percentage share of pores with diameters ranging from 0.25 µm to 0.30 µm for GO and MWCNT coated membranes or 0.30 µm and more for SWCNT. This is due to the partial blocking of membrane pores with ODTS molecules and particles of carbon compounds. It should also be noted that the changes in the percentage share of the different diameter ranges varied according to the modifying compound used. This may be due to the different sizes of these compounds and their ability to settle on the surface and in the membrane pores. Moreover, the test of the contact angles shows that the modified membranes have less wettability than the unmodified one, which also reduces permeability. It should be noted that the diameter size distribution did not change greatly for the modified membranes as compared to the unmodified ones. The modified membranes can still be classified as microfiltration membranes.

The results presented above show that the selected modifications of the membranes led to an increase in the efficiency of removing PPCPs from water from a few percent up to even 50% (GO_1_60) and over 100% (MWCNT_1_60) compared to the unmodified membrane. It is difficult to perform a comparative analysis of our results with the literature ones due to the differences in the membranes used, modification agents used and differences in the tested PPCPs. However, it can be noticed that the results presented in the literature indicate that the modification can significantly improve the properties of the membranes in relation to PPCPs, even by 35% [26]. However, other reports indicate no significant improvement [28] or even deterioration of the examined properties [27]. In the papers where 100% removal of the tested PPCPs was achieved [29], different membrane techniques were used than in this study. Table 4 presents a comparison of performance of different membranes used for PPCP removal. It also includes selected results from this paper. It can be seen that PPCP rejection by our membrane is satisfactory and the permeate flux is very high.

## 4. Conclusions

Ceramic-based membranes modified by carbon compounds demonstrate the ability to adsorb pharmaceuticals from water streams that are in contact with the membrane. Ceramic membranes modified by MWCNT are characterized by high, as for microfiltration membranes, retention of tetracycline, namely, 45.4%. Modifications with other carbon compounds like SWCNT and GO did not lead to the satisfactory results. Providing membranes with adsorption properties does not significantly impair the filtration properties of the membranes. The permeate flux is still high (520 L·h·m^−2^·bar^−1^). Therefore, such membranes can be used in an integrated filtration–adsorption process. At the same time, selected components will be retained by the membrane and pharmacological compounds that, due to their size, cannot undergo retention on a porous membrane will be adsorbed on the membrane surface. The membranes developed in this study can be used successfully in the processes of removing Pharmaceutical and Personal Care Products (PPCPs) from the aquatic environment.

## Figures and Tables

**Table 1:** Modification process parameters of the test membranes in the stationary system.

Membrane Type	ODTS Solution Concentration [%]	Modification Time in Carbon Compound Suspension [min]
UNMOD	0	0
ODTS_0.5	0.5	0
ODTS_1	1	0
ODTS_5	5	0
MWCNT_0.5_10	0.5	10
MWCNT_0.5_30	0.5	30
MWCNT_0.5_45	0.5	45
MWCNT_0.5_60	0.5	60
SWCNT_0.5_10	0.5	10
SWCNT_0.5_30	0.5	30
SWCNT_0.5_45	0.5	45
SWCNT_0.5_60	0.5	60
GO_0.5_10	0.5	10
GO_0.5_30	0.5	30
GO_0.5_45	0.5	45
GO_0.5_60	0.5	60

**Table 2:** Modification process parameters of the test membranes in the flow-through system.

Membrane Type	ODTS Solution Concentration [%]	Modification Time in Carbon Compound Suspension [min]
UNMOD	0	0
ODTS_0.5	0.5	0
MWCNT_0.5_60	0.5	60
MWCNT_1_60	1	60
SWCNT_0.5_60	0.5	60
SWCNT_1_60	1	60
GO_0.5_60	0.5	60
GO_1_60	1	60

**Table 3:** Concentration of tetracycline in permeate and retentate during the process.

Process Time	UNMOD		MWCNT_1_60		GO_1_60	
[min]	*c_N_* [g/dm^3^]	*c_P_* [g/dm^3^]	*c_N_* [g/dm^3^]	*c_P_* [g/dm^3^]	*c_N_* [g/dm^3^]	*c_P_* [g/dm^3^]
0	0.033	0.033	0.032	0.032	0.033	0.033
1	0.033	0.033	0.032	0.032	0.033	0.033
5	0.032	0.031	0.029	0.025	0.030	0.028
10	0.030	0.031	0.026	0.025	0.029	0.028
15	0.029	0.030	0.024	0.024	0.028	0.027
30	0.027	0.029	0.021	0.023	0.026	0.026
60	0.027	0.027	0.021	0.020	0.025	0.025
90	0.026	0.026	0.019	0.018	0.024	0.023

**Table 4:** PPCP removal by membranes—comparison.

Membrane Material	Membrane Process	PPCP	Flux L·h^−1·^m^−2·^bar^−1^	Retention %	Mechanism	Reference
Al_2_O_3_ + MWCNT	MF	Tetracycline	520	45.4	Adsorption/filtration	This work
PES + CSMM	UF	Ibuprofen	29.0	12.57	Adsorption/filtration	[28]
PES + GO	UF	Ibuprofen	5.27	44.9	Adsorption/filtration	[29]
PA + GO	NF	Norfloxacin	12.78	53.32	Filtration/adsorption	[31]
CA	NF	Sulfamethazine	2.87	85.2	Filtration	[26]
CA + CSMM	NF	Sulfamethazine	1.85	84.1	Filtration	[26]
CA + LSMM	NF	Sulfamethazine	1.54	78.6	Filtration	[26]

CA—cellulose acetate, CSMM—charged surface modifying macromolecule, GO—graphene oxide, LSMM—hydrophilic surface modifying macromolecule, MF—microfiltration, MWCNT—multi-walled carbon nanotubes, NF—nanofiltration, PA—polyamide, PES—poly(ether sulfone), UF—ultrafiltration.
